# Effects of Chilling and Anoxia on the Irradiation Dose-Response in Adult *Aedes* Mosquitoes

**DOI:** 10.3389/fbioe.2022.856780

**Published:** 2022-05-02

**Authors:** H. Yamada, H. Maiga, C. Kraupa, W. Mamai, N. S. Bimbilé Somda, A. Abrahim, T. Wallner, J. Bouyer

**Affiliations:** ^1^ Insect Pest Control Laboratory, Joint FAO/IAEA Centre of Nuclear Techniques in Food and Agriculture, International Atomic Energy Agency, Vienna, Austria; ^2^ Department for Insect Biotechnology, Justus-Liebig-University Gießen, Gießen, Germany; ^3^ Food and Environmental Protection Laboratory, Joint FAO/IAEA Centre of Nuclear Techniques in Food and Agriculture, International Atomic Energy Agency, Vienna, Austria

**Keywords:** induced sterility, mosquito, irradiation, *Aedes aegypti*, *Aedes albopictus*

## Abstract

The success of the sterile insect technique (SIT) relies on the achievement of high levels of sterility and mating success of the factory-reared sterile males and thus their biological quality, which can be enhanced by the reduction of stress factors encountered during rearing, handling, and irradiation procedures. The achievement of consistent sterility levels requires reliable and standard irradiation protocols. Additionally, mosquito adults require immobilization prior to, and during irradiation to increase processing efficiency and to avoid physical damage caused by movement in restricted space. Common methods for immobilization include chilling and anesthetics such as nitrogen. Here we assessed the effects of chilling and exposure to nitrogen on the irradiation dose-response of *Aedes* mosquitoes, and their downstream effects on some male quality parameters including longevity and flight ability. We found that chilling does not incur damage in the insects in terms of longevity and flight ability when chilling duration and temperature are carefully controlled, and a recovery phase is provided. Irradiation in nitrogen shows high radioprotective effects during irradiation, resulting in reduced induction of sterility. Overall, longevity of males can be improved by irradiating in anoxia, however the exposure to nitrogen itself comes with negative impacts on flight ability. The results reported here will assist in the standardization and optimization of irradiation protocols for the SIT to control mosquito populations of medical relevance.

## 1 Introduction

The sterile insect technique (SIT) (Dyck et al., 2021) is a biological insect population control tactic that reduces the dependence of insecticides and thus agrees with present day concerns regarding human health and the environment. The SIT concept has been in existance since the 1930’s, and has been implemented against various crop pests with huge success since the 1950’s. It was first implemented against mosquitoes in the 1960’s with varying results (Dame et al., 2009), however, the technique has more recently regained interest in the fight against malaria, and in response to the Zika virus outbreaks in 2015. Following increasing demands from Member States, the Insect Pest Control Laboratory of the Joint FAO/IAEA Centre of Nuclear Techniques in Food and Agriculture has been developing the SIT package for select disease transmitting mosquito species, in particular *Aedes aegypti* and *Ae. albopictus* (the main vectors of dengue, Zika, chikungunya among other arboviruses) and *Anopheles arabiensis*, an important vector of malaria. Great progress has been made for each component of the SIT, resulting in the development of equipment, methods and guidelines for mass rearing, sex separation, irradiation, packing, transportation, quality control, release methods, and field trials. The most notable achievements of the past decade are reviewed in (Vreysen et al., 2021).

The success of the SIT relies on the reliable induction of sterility in the target insect population by releasing mass produced sterile males into the field, where they must outcompete wild counterparts to secure mating which results in no offspring. For this, dependable irradiation protocols are required to ensure constant, and high levels of induced sterility, whilst maintaining the highest possible quality in the sterile insects. The irradiation of medically important mosquito species in the frame of the SIT requires the males to be near to fully sterile both to avoid a risk of replacement of the target population (WHO and IAEA, 2020), and to ensure a maximal efficiency of the sterile males given the high reproduction rate of these species (Aronna and Dumont, 2020). This can be achieved by exposing pupae or adults to ionizing radiation- usually in gamma-ray irradiators (Helinski et al., 2009 and references within), and more recently, in X-ray irradiators (Mastrangelo et al., 2010; Yamada et al., 2014; Du et al., 2019; Zheng et al., 2019), and possibly with industrial accelerators producing electron beams (Balestrino et al., 2016), although these devices are currently used mainly in phytosanitary applications (Smittle et al., 1991; Dohino et al., 1997; Todoriki et al., 2006; Koo et al., 2012).

Although there is a high degree of reliability when achieving expected sterility levels by exposure to a known dose according to dose-response studies, some physical factors influence the dose response in mosquitoes, and biological factors also affect their general sensitivity to radiation. Some of these factors have been studied more frequently, such as the effects of life stage, gender and pupal age (Wakid et al., 1976; Helinski et al., 2006, 2009; Balestrino et al., 2010; Akter and Khan, 2014) wheras very few, or only very old reports exist for the evaluation of others factors, such as effects of hypoxia or anoxia, temperature, and dose rate during radiation exposure (Hallinan and Rai, 1973; Curtis, 1976; El-Gazzar et al., 1983; Ernawan et al., 2017; Zhang et al., 2020).

More recently, a series of experiments to assess the impacts of several biological and physical factors (e.g., strain geographical origin, pupal age pupal size, atmospheric conditions) on dose-reponse in mosquitoes were conducted (Yamada et al., 2019; Yamada et al., 2020) with the aim to develop standardized protocols for the irradiation of mosquito pupae (FAO/IAEA, 2019). However; standardizing irradiation protocols for pupae is difficult, especially in practical terms in large-scale SIT programmes for the following reasons: Pupal age is an important factor that significantly impacts dose-response (Balestrino et al., 2010; Yamada et al., 2019). Although guidelines exist for the optimization of larval rearing for synchronized pupae development (FAO/IAEA, 2020), it is in reality unrealistic to narrow the pupation window to 16 h or shorter, to ensure that all pupae are aged 30 h or older during the irradiation process. Also, timing the pupation so that the collection, sexing and irradiation can occur during daytime working hours is another challenge. Irradiating mixed age batches is not recommended, as irradiating younger pupae can negatively affect adult quality (Balestrino et al., 2010), and over-dosing (as younger pupae require less dose) would further exacerbate this. Conversely, irradiating younger pupae at an optimal dose (to achieve >99% sterility) is possible, however the risk remains that older pupae would be under-dosed, leading to potentially releasing sub-sterile males, in addition to males with diminished quality, thereby compromising success of the otherwise effective SIT. Additionally, and equally problematic is that it is difficult, if not impossible to control the atmospheric conditions surrounding pupae during irradiation in bulk. For mass irradiation at the pupal stage, the pupae would need to be placed in sufficient water within the irradiation canister to provide buoyancy to avoid the pupae at the bottom being crushed. However, this creates a hypoxic environment as pupae submerged in water continue to respirate through their cuticle and quickly deplete the surrounding water of dissolved oxygen (Yamada et al., 2020). As hypoxia reduces irradiation effects, the irradiation of pupae in water results in differential levels of sterility within the sample (Yamada et al., 2020), therefore this method for irradiation cannot be reliable unless, again, the full cohort is significantly overdosed. Apart from quality costs of over-dosing, pupae exposed to hypoxia suffer additional stress and loss in quality. Large numbers of pupae can also be irradiated without water in monolayers, however, pupae are still closely packed and pockets of hypoxia still occur within the sample (Louis Clement Gouagna personal communication) resulting in a proportion of pupae maintaining unacceptable levels of fertility. Drying pupae and spreading them in a manner that would avoid these issues is simply not practical at large scale and is expected to incur detrimental levels of stress to the pupae.

For these reasons, the irradiation at adult stage could be a more practical and reliable option for the bulk sterilization of mosquitoes. Most notably, water, and thus hypoxia would no longer be a variable factor. Perfectly synchronized larval rearing (to achieve pupation within a 24 h window) would also no longer be as critical (and limiting) issue, significantly easing the practicality and efficiency of the irradiation process. However, to irradiate adult mosquitoes in bulk, these require immobiliaztion by either chilling (Culbert et al., 2019; Zhang et al., 2020) or treatment with anesthetics, such as in nitrogen, carbon dioxide, argon, chloroform, desflurane, or other alternative chemicals.

To verify the notion that standardizing irradiation for adult mosquitoes is feasible, we investigated some factors that may affect dose-response in adult male mosquitoes in comparison to pupae. Previous reports by Helinski et al. (Helinski et al., 2006) and Du et al. (Du et al., 2019) have shown that in both *Anopheles arabiensis* and *Aedes albopictus* respectively, adults are slightly more radio sensitive than old pupae, although the difference was generally not statistically significant. As no recent reports cover the comparative radiosensitivity of adults and old pupae in *Ae. aegypti*, we first studied the dose response curves of both life stages in this species, and then assessed the effects of ambient temperature (chilling), and anoxia in *Ae. albopictus* adults.

## 2 Materials and Methods

### 2.1 Mosquito Strains and Rearing

Standard laboratory reference strains of *Ae. aegypti* and *Ae. albopictus* (FAO/IAEA, 2017, 2020) were used for all experiments. The *Aedes* strains have been maintained following the “Guidelines for Routine Colony Maintenance of *Aedes* mosquitoes” (FAO/IAEA, 2017).

### 2.2 Irradiation and Dosimetry

Radiation treatments were performed in a Gammacell 220 (Nordion Ltd., Kanata, Ontario, Canada), which had a dose-rate of 68 Gy/min during the temperature experiment (Section 2.4), and 65 Gy/min during the anoxia experiment (Section 2.5).

The dosimetry system used to verify the dose received by the samples was based on Gafchromic HD-V2 and MD-V3 film (Ashland Advanced Materials, Bridgewater NJ, United States) following the IAEA protocol (IAEA, 2004). Three films of either HD film (for doses >50 Gy) or MD film (for doses <50 Gy) were packed in small (2 × 2 cm) paper envelopes and placed directly above and below the mosquito samples. Films were read with an optical density reader after 24 h of development.

A diagnostic dose of 45 Gy was applied for most experiments, expecting to achieve around 95% sterility, to avoid 0 hatch results that cannot be usefully compared between treatments.

### 2.3 Assessing the Dose Response Curve for Pupal and Adult Stages of *Aedes aegypti*

*Aedes aegypti* were selected for this study as direct comparisons of pupal and adult radiosensitivity have not yet been reported in this species, contrary to *Ae. albopictus* (Du et al., 2019) and *An. arabiensis* (Helinski et al., 2006).

The doses for the dose-response curves for adult versus pupae of *Ae. aegypti* were selected according to the expected dose required to induce 50–100% sterility: 20, 55, 70, and 90 Gy.

*Aedes aegypti* eggs from one egg batch were collected and split in half to be hatched in two hatch events, 2 days apart (one for collecting adults, and one for collecting pupae for irradiation at the same time).

Adult males that emerged within an 8 h window were collected, batched in groups of 30, and kept in 15 × 15 × 15 cm Bugdorm® cages (MegaView Science Co. Ltd., Taichung 40762, Taiwan) until the following day when they were transferred to, and irradiated in small 2 cl plastic cups closed with a sponge. At the time of irradiation, the adults were 24–32 h old.

Pupae from the same cohort were collected in 4-h windows to ensure uniform pupal age of 40–44 h. We chose this age group as this represents the last hours before they begin to emerge into adults and are most radioresistant at this stage. The pupae were sexed based on pupal size dimorphism using a glass pupal sorter (Focks, 1980) and sex was verified under a stereomicroscope. Males were kept for treatment and females were placed in individual tubes for emergence to ensure virginity for later mating. Male pupae were counted into batches of 30 and were placed inside 2 cl plastic cups with excess water removed for irradiation.

Both the pupae and adults in each technical repetition were irradiated at the same time. Two biological repetitions and three technical repetitions were performed for all doses. Controls received the same handling but were not irradiated.

#### 2.3.1 Assessment of Induced Sterility

Following irradiation, the male adults were placed in a 15 × 15 × 15 cm Bugdorm® cage, and pupae were placed in cups with water in separate cages for emergence. Thirty virgin females were added to each cage when the adults reached 2 days of age and were allowed to mate for 3 days before they were provided with 2 bloodmeals on consecutive days (days 6 & 7 post-emergence). Oviposition cups containing water and germination papers were added to each cage on day 8 for *en masse* egg collection (on days 9 & 10 post-emergence) following routine rearing protocols (FAO/IAEA, 2017). Egg papers were collected, matured (slow-dried over 4 days) and stored for 10 days before hatching. The total number of hatched and un-hatched eggs were counted using a stereomicroscope. Any non-hatched eggs were either opened with a dissection needle, or if many, were bleached to determine the fertility status (FAO/IAEA, 2019).

### 2.4 Effects of Chilling on Pupae and Adult Radiosensitivity, Flight Ability and Longevity in *Aedes albopictus* Irradiated as Adults

#### 2.4.1 Dose-Response

As for the previous experiment, *Ae. albopictus* eggs from one egg batch were collected and split in half to be hatched in two hatch events, 2 days apart.

Adult males that emerged within an 8 h window were collected, batched in groups of 30, and were kept in 15 × 15 × 15 Bugdorm cages until the following day. The cages were then either kept at room (insectary) temperature (27° ± 2°C) (“rm temp”) or were placed in a cold room for knock down at 5°C for 5 min, and then in a climatic chamber at 7°C (“chilled”) for 1 h prior to the irradiation event. The treatments were thus either Control rm temp, or 45 Gy rm temp, or control chilled, or 45 Gy chilled. The adult males for the irradiation treatment were then transferred to 2 cl plastic cups closed with a sponge and were taken to the irradiator in Styrofoam boxes; the chilled males were kept in the cool box at 7°C until placed inside the GC220 irradiator chamber and irradiated in same small 2 cl plastic cups closed with a sponge. At the time of irradiation, the adults were 2 days old.

Pupae from the same cohort were collected in an 8-hour window to ensure that all pupae were at least 36 h old during irradiation. The pupae were sexed based on pupal size dimorphism using a glass pupal sorter (Focks, 1980) and sex was verified under a stereomicroscope. Males were kept for treatment and females were placed in individual tubes for emergence to ensure virginity for later mating for both the adults and pupae treatment groups. Male pupae were counted into batches of 30 and were placed inside 2 cl plastic cups. The samples were subjected to the same treatments as described for adults above. The treatments were thus either Control rm temp, or 45 Gy rm temp, or control chilled, or 45 Gy chilled. Before irradiation, excess water was removed from the cups holding pupae. Two biological repetitions with each 3 technical repetitions were performed for each treatment.

Egg hatch rates were assessed as described in the above section “Assessing the dose response curve for pupal and adult stages following irradiation in a GC220/2.3.1 Assessment of induced sterility”.

#### 2.4.2 Longevity Under Mating Stress

Three of each treatments group and controls each, for both “adults” and “pupae” were kept in the cages post mating and oviposition to follow the longevity of the males. Dead males were counted and removed at least 4 times per week until all males were dead.

#### 2.4.3 Flight Ability

One hundred male adults per treatment (rm temp or chill) and per repetition were collected from the same cohort and irradiated as described in the above section “dose response”.

After irradiation, adults were allowed to recover for 2 days before they were taken to the flight test devices. Each batch of 100 males were placed inside the flight tubes for a duration of 2 h. Escaped and non- escaped adults were then counted as described by Culbert et al. (Culbert et al., 2018). Two repetitions with each 2 technical repetitions were completed.

### 2.5 Effects of Anoxia on Adult Dose-Response*,* Flight Ability and Longevity in *Aedes albopictus*

#### 2.5.1 Dose-Response

**FIGURE 1:**
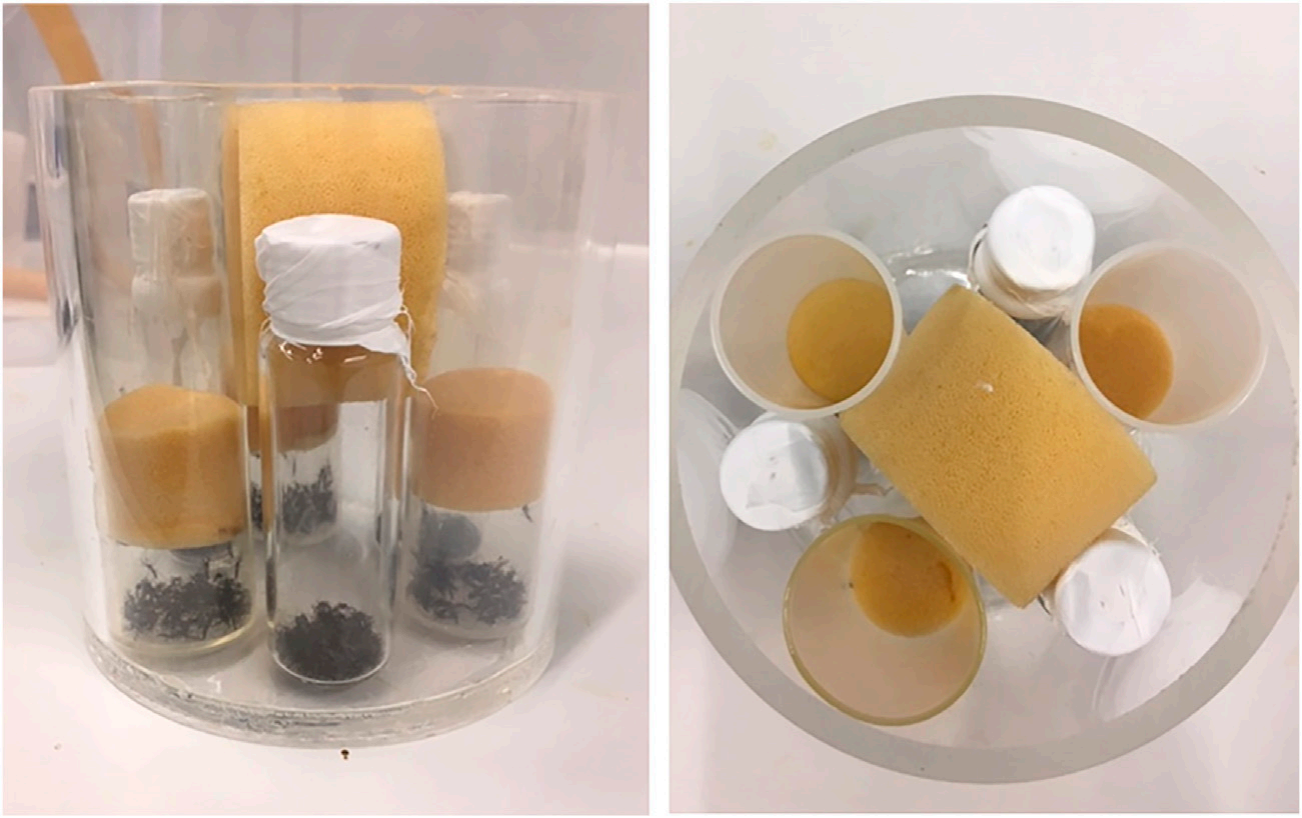
Irradiation set-up for adults irradiated in nitrogen or in air. One biological repetition with each three technical repetitions were irradiated simultaneously for each treatment, within a 5 mm thick PMMA container. The container was placed on a Styrofoam step so that all samples were in the middle of the irradiation chamber.

Adult *Ae. albopictus* males that emerged within an 8 h window were collected, batched in groups of 20–30, and were kept in 15 × 15 × 15 cm Bugdorm cages until the following day. The batches of adult males for the “normoxic treatment” were then transferred to plastic “*Drosophila*” tubes (9 cm height × 2.7 cm diameter) closed with a sponge. The sponges were pushed down before irradiation so that the samples were in a similar position to the adults immobilized with nitrogen (Figure 1). Adult batches for the “anoxic treatment” were placed in gas tight glass head space vials (20 ml) with screw tops with PTFE/silicon septum (Merck KGaA, Darmstadt, Germany), additionally sealed with PTFE Thread Seal Tape (Sigma-Aldrich, United States). The oxygen was then replaced by nitrogen by adding nitrogen *via* a syringe needle (a second needle was inserted for outgoing gas), for 10 s, until all adult mosquitoes were immobile, and the 2 syringe needles were removed. Both anoxic and normoxic groups (3 technical repetitions each) were irradiated at 45Gy simultaneously (in alternating positions) in a 12 cm diameter PMMA container, in the GC220 irradiator (Figure 1). Three biological repetitions from different cohorts (with each 3 technical repetitions) were performed in total. At the time of irradiation, the adults were 2 days old.

Induced sterility was assessed as described in the above section “Assessing the dose response curve for pupal and adult stages following irradiation in a GC220/2.3.1 Assessment of induced sterility”.

#### 2.5.2 Flight Ability

One hundred male adults per treatment (normoxia (oxygen) or anoxia (nitrogen), irradiated at 45 Gy, and non-irradiated controls) and per repetition were collected from the same cohort and irradiated as described in the above section “dose-response”.

Flight tests were performed as described in the above section “Section 2.4 and Section 2.4.3”*.* Two repetitions with each 3 technical repetitions were completed.

#### 2.5.3 Longevity Under Mating Stress

All treatments groups and controls were kept in the cages post mating and oviposition to follow the longevity of the males. Dead males were counted and removed at least 4 times per week until all males were dead. Three repetitions were done for all treatment groups and controls for both treatment groups irradiated as pupae and as adults.

#### 2.5.4 Longevity Following High Doses- Males Only

As little difference was seen following the previous longevity experiments, and mating stress is known to decrease survival in males, an additional experiment was added to assess the effects of anoxia on sterile male longevity, without the added factor of mating stress. For this, additional batches of 30 males were irradiated in either normoxia (oxygen) or anoxia (nitrogen) as described in the previous section. All males were over-dosed at 90 Gy (beyond the fully sterilizing dose) in the GC220 as described above. The males were then returned to 15 × 15 × 15 cm Bugdorm cages and dead males were counted and removed at least 4 times per week until all males were dead.

### 2.6 Statistical Analysis

All statistical analyses were performed in R (version 4.1.0) using RStudio (RStudio, Inc. Boston, MA, United States, 2016). Generalized Linear Mixed Models (GLMM, lme4 package) were used with the appropriate distribution family.

To analyze the dose response curve of pupae versus adults for *Ae. aegypti*, a binomial GLMM fit by maximum likelihood (Laplace Approximation) was used for egg hatch rates considered as response variable, life stage (2 levels: pupae and adults), irradiation log (dose) (4 levels: 20, 55, 70 and 90 Gy) and their interaction were considered as fixed effects and the repetition as a random effect.

For the effects of chilling on pupae and adult radio-sensitivity in *Ae. albopictus*, a binomial GLMM was also used with egg hatch rates as response variable, treatment (2 levels: room, chilling), life stage (2 levels: pupae and adults), irradiation dose (2 levels: 0 and 45 Gy) and their interaction considered as fixed effects and repetition nested with technical repetition as a random effect.

Similarly, male flight ability data was analyzed as response variable, treatment (4 levels: Chilled/room temperature, irradiated/non-irradiated; or anoxia/normoxia and irradiated/non-irradiated) as fixed effect and the repetition nested with technical repetition as a random effect considering each specific experiment.

Mixed Effects Cox Models (“coxme” function in ‘survival’ package) fit by maximum likelihood with mosquito time to death as response variable, treatment (4 levels: chilled, room temperature, irradiated, non-irradiated; or 3 levels: anoxia, normoxia, non-irradiated control) and their interaction as fixed effects and repetition as random effect, were used to analyze the survival of mosquitoes following the treatment in each specific experiment. Survival graphs were built using the packages “survival,” “ggplot2,” and “ggpubr”.

The full models were checked for overdispersion using Bolker’s function (Bolker and R Development Core Team, 2020) (in package bblme). The best model was chosen based on the lowest AICc s and models were simplified using the stepwise removal of terms, followed by likelihood ratio tests (LRTs) when appropriate. Multiple comparisons using the “emmeans” function (in package “emmeans”) (https://github.com/rvlenth/emmeans) were performed between the levels where significant differences were found. A *p*-value of less than 0.05 was used to indicate statistical significance in all cases.

## 3. Results

### 3.1 Dosimetry

The dosimetry confirmed that all doses received laid within a 5% error range.

### 3.2 Assessing the Dose Response Curve for Pupal and Adult Stages of *Aedes aegypti*

**FIGURE 2:**
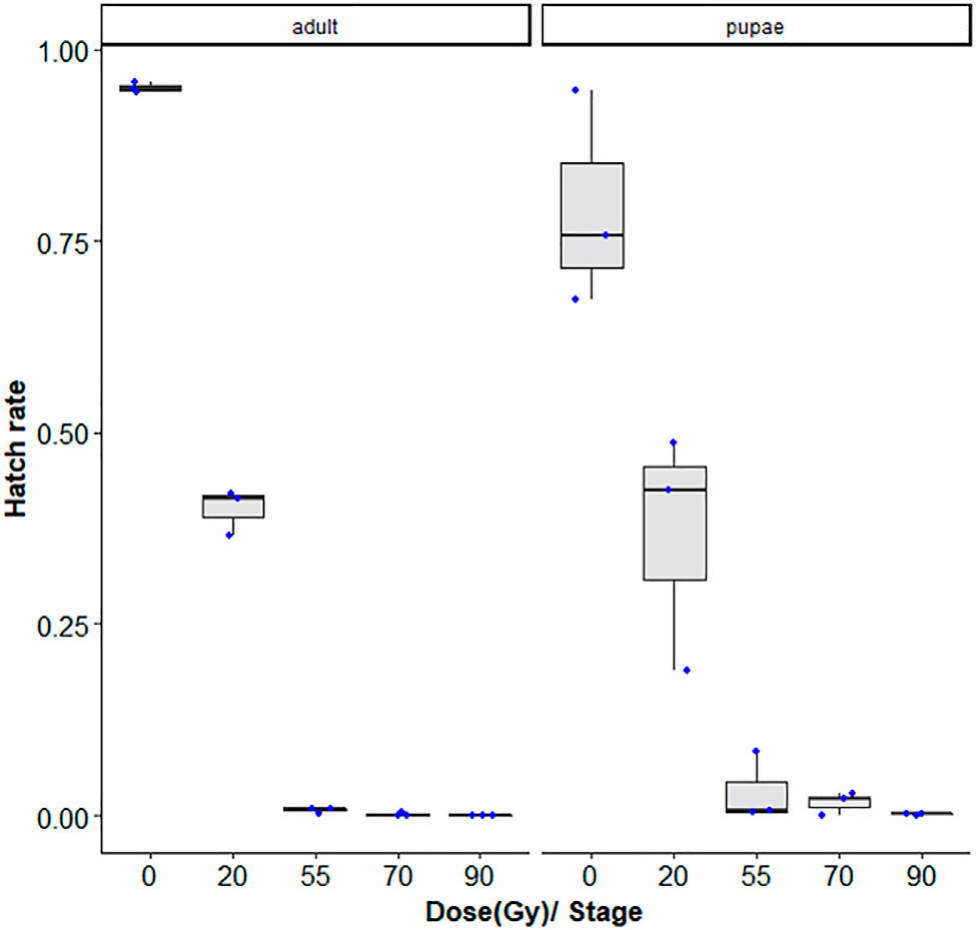
Dose-response shown as egg hatch rate of *Aedes aegypti* pupae vs. adults. The boxplot shows the median, and upper and lower quartiles. The dots indicate the values obtained for each repetition.

As expected, the hatch rate reduced signficantly with the dose (GLMM: χ^2^ = 2,589, df = 2, *p* < 0.001). For 20, 55, 70, and 90 Gy doses tested, adults were more radiosensitive than the late stage pupae, with <1–∼3% lower fertiliy levels following radiation exposure (GLMM: χ^2^ = 52.685, df = 2, *p* < 0.001, Supplementary Table S1). There was also a higher degree of variation observed between repetitions for the pupae samples (Figure 2).

### 3.3 Effects of Chilling on Pupae and Adult Radiosensitivity, Flight Ability and Longevity in *Aedes albopictus* Irradiated as Adults

#### 3.3.1 Dose-Response

**FIGURE 3:**
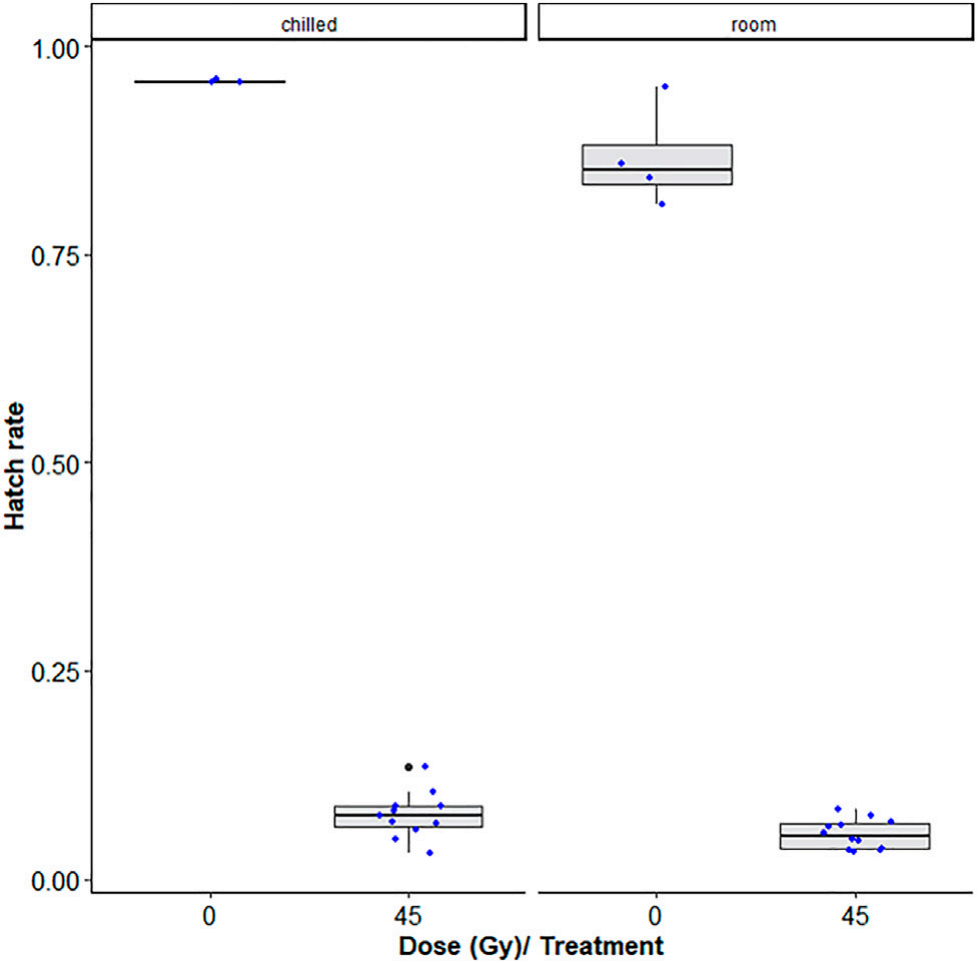
Dose-response shown as egg hatch rate of *Aedes aegypti* pupae vs. adults. The boxplot shows the median, and upper and lower quartiles. The dots indicate the values obtained for each repetition.

Table 1 shows that room temperature led to lower hatch rates as compared to chilling, i.e., chilling led to decreased induced sterility (GLMM: χ^2^ = 6.4454, df = 1, *p* = 0.0111, Figure 3) while no difference was observed between pupae and adult *Ae. albopictus* mosquito stages (χ^2^ = 3.1432, df = 1, *p* = 0.07625)*.* Additionally, irradiation dose of 45Gy significantly reduced the egg hatch rates (χ^2^ = 809.1845, df = 1, *p* < 0.001, Figure 3).

**TABLE 1:** Fixed effects of chilling on pupae and adult radiosensitivity.

	Estimate	Std. Error	z value	Pr (>|z|)
(Intercept)	2.0095	0.1826	11.005	<2e-16 ***
Room temperature	−0.3408	0.1342	−2.539	0.0111 *
Pupae stage	0.2332	0.1315	1.773	0.0762
Dose45Gy	−4.6976	0.1651	−28.446	<2e-16 ***

Signif. codes: 0 “***” 0.001 “**” 0.01 “*” 0.05 “.” 0.1 “ ” 1.

#### 3.3.2 Effects of Chilling on Flight Ability

**FIGURE 4:**
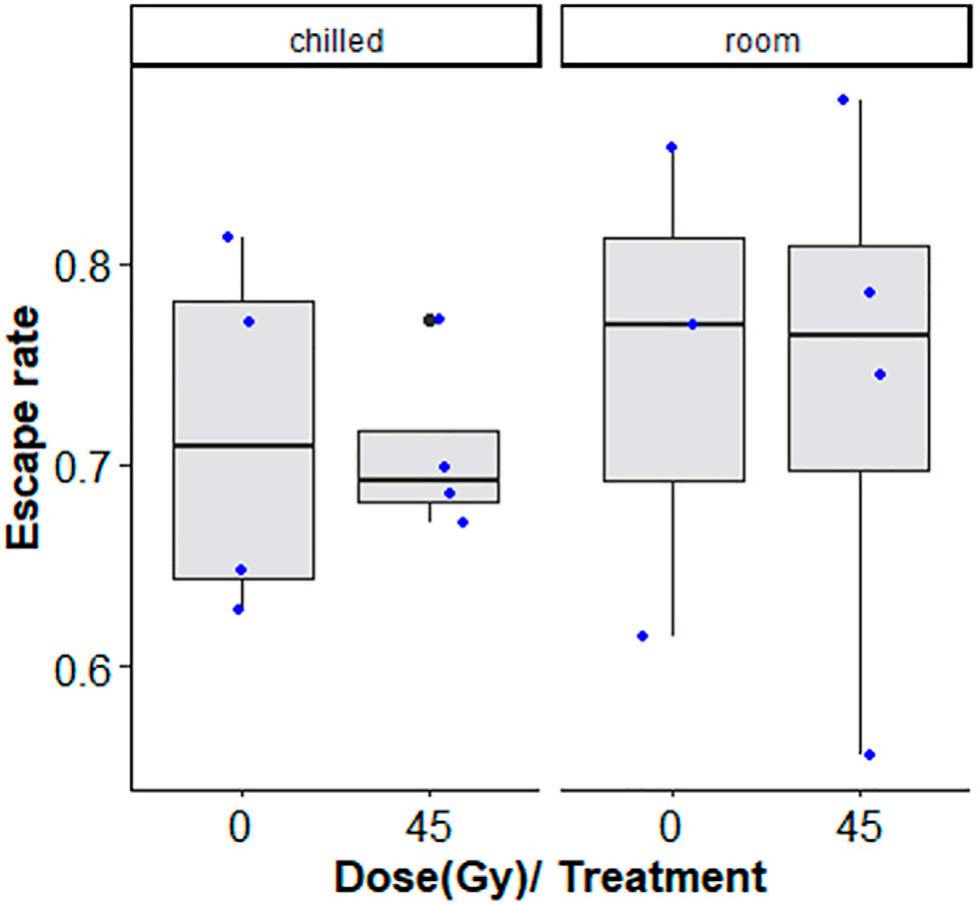
Flight ability of chilled versus non-chilled, sterile *Aedes albopictus* male adults irradiated at 45 Gy or not irradiated The boxplot shows the median, and upper and lower quartiles. The dots indicate the values obtained for each repetition.

Chilling for 1 h at 7°C, with or without irradiation at 45 Gy followed by 2-day-recovery had no negative effects on flight ability (Figure 4; Table 2).

**TABLE 2:** Fixed effects of chilling and irradiation on flight ability in *Aedes albopictus males*.

	Estimate	Std. Error	z value	Pr (>|z|)
(Intercept)	0.85361	0.09806	8.705	<2e-16 ***
Room temperature	0.15859	0.10535	1.505	0.132
Dose45Gy	0.04216	0.09957	0.423	0.672

Signif. codes: 0 “***” 0.001 “**” 0.01 “*” 0.05 “.” 0.1 “ ” 1.

#### 3.3.3 Effects of Chilling on Male Longevity

**FIGURE 5:**
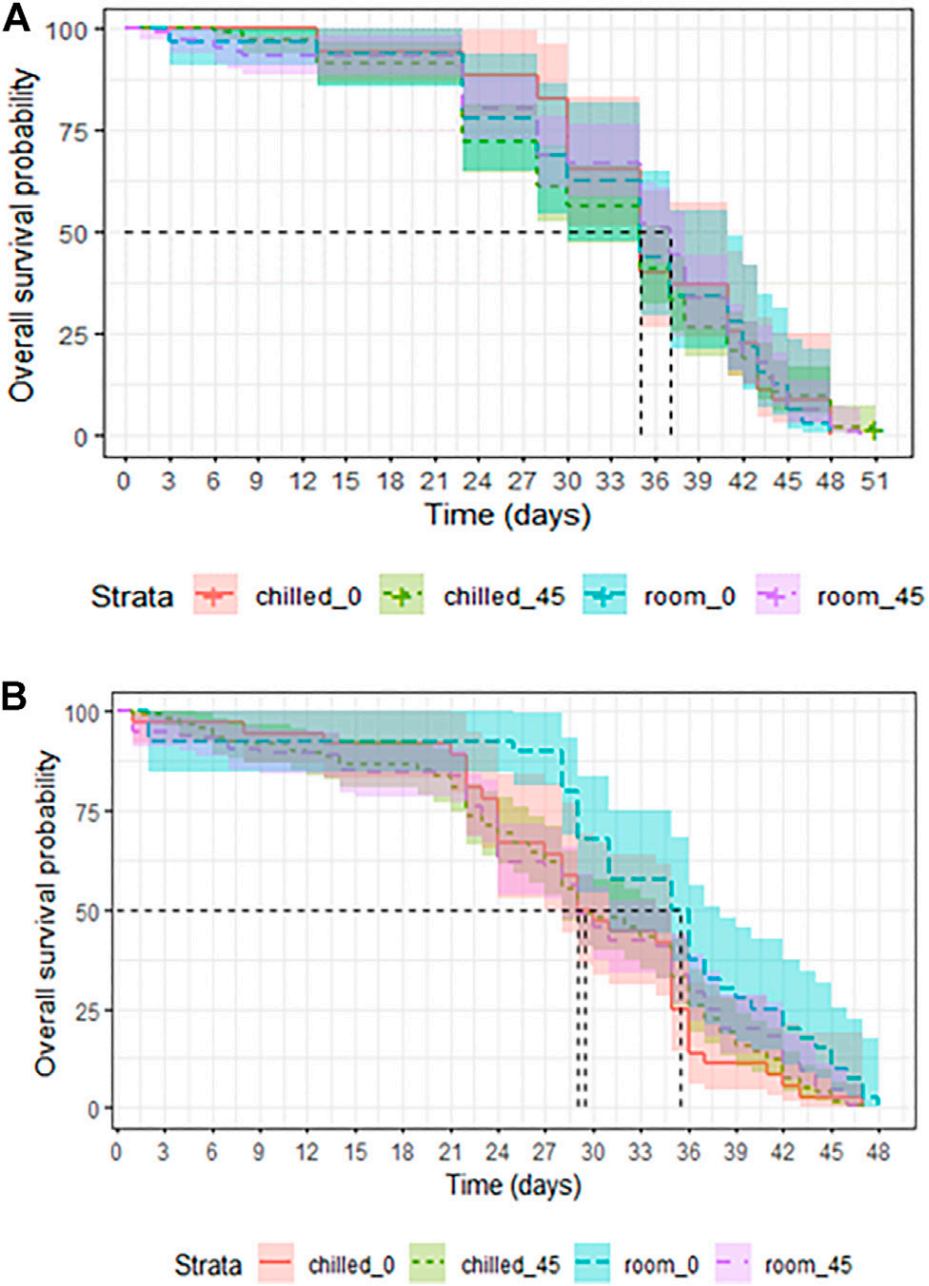
Longevity of chilled versus non-chilled *Aedes albopictus* males irradiated at 45 Gy as either pupae **(A)** or as adults **(B)**, compared to non-irradiated controls.

In adults that were chilled and/or irradiated as late pupae, survival was not affected by chilling (LRT: χ^2^ = 0.1582, df = 1, *p* = 0.6908, Table 3), nor by irradiation with 45Gy (LRT: χ^2^ = 0.0006, df = 1, *p* = 0.9383, Table 3). Adults that were chilled and irradiated as pupae also lived as long as untreated controls (LRT: χ^2^ = 0.224, df = 1, *p* = 0.6317, Table 3 and Figure 5A).

**TABLE 3:** Fixed coefficients of the effects of pupae chilling on male *Ae. albopictus* longevity.

	Coef	exp (coef)	se (coef)	z	*p*
Dose	0.001195	1.001196	0.004363	0.27	0.78
Doom temperature	0.05391	1.055389	0.24482	0.22	0.83
Dose:room temperature	−0.003	0.997009	0.006248	−0.48	0.63

Signif. codes: 0 “***” 0.001 “**” 0.01 “*” 0.05 “.” 0.1 “ ” 1

In mosquitoes that were chilled and/or irradiated at the adult stage, chilling had no negative effect on the overall longevity of adults (LRT: χ^2^ = 2.947, df = 1, *p* = 0.08603, Table 4). Irradiation at 45 Gy also did not affect overall longevity as compared to non-irradiated adults (LRT: χ^2^ = 2.2893, df = 1, *p* = 0.1303, Table 4). Adults that underwent chilling plus irradiation at 45 Gy were marginally negatively impacted as compared to untreated controls (LRT: χ^2^ = 3.39, df = 1, *p* = 0.06559, Figure 5B). Chilling, and combined chilling and irradiation treatments reduced the median survival from the 35.5 (31-38, 95%CI) days in the control group to 29.0–29.5 (28-35, 95%CI) days (Supplementary Table S2), however, the difference was not significant.

**TABLE 4:** Fixed coefficients of the effects of adult chilling on male *Ae. albopictus* longevity.

	coef	exp (coef)	se (coef)	z	*p*
Dose45Gy	0.210645	1.234474	0.138563	1.52	0.13
Room temperature	−0.19772	0.820597	0.11508	−1.72	0.086

Signif. codes: 0 “***” 0.001 “**” 0.01 “*” 0.05 “.” 0.1 “ ” 1.

### 3.4 Effects of Anoxia on Adult Dose-Response, Flight Ability and Longevity in *Aedes albopictus*

#### 3.4.1 Dose-Response

**FIGURE 6:**
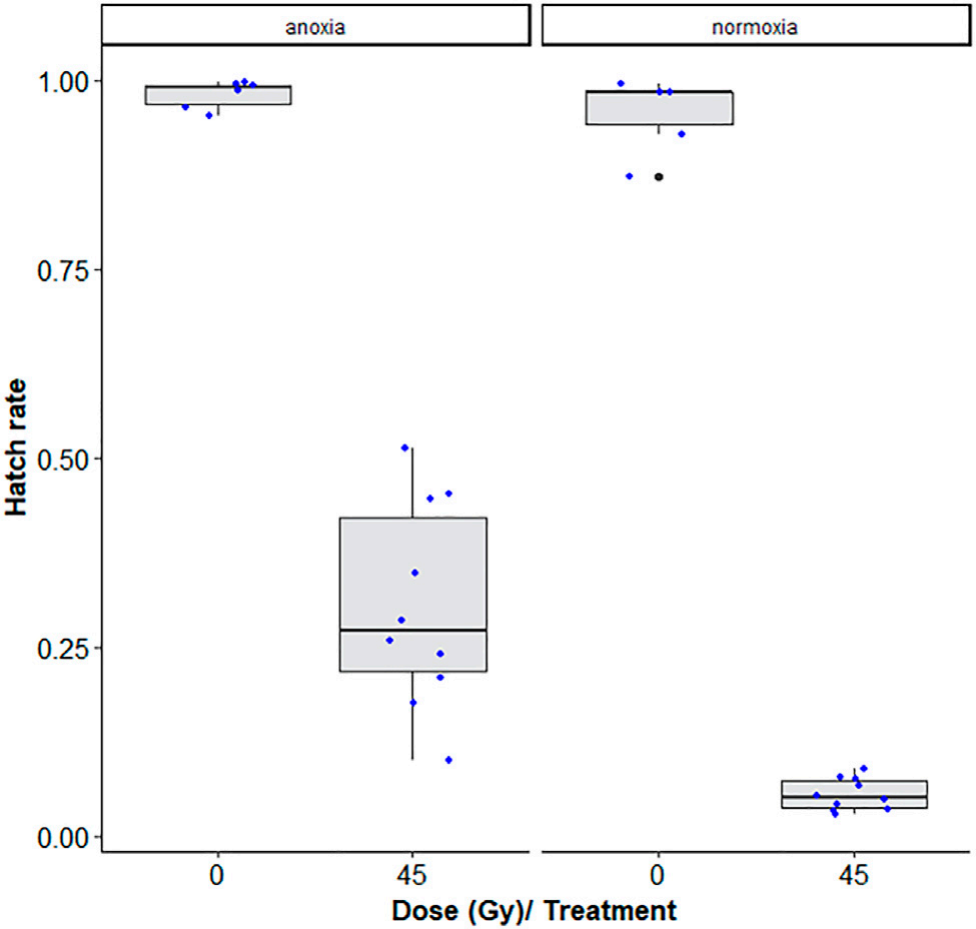
Egg hatch rates of *Aedes albopictus* males irradiated at 45Gy in normoxia (O_2_) vs. anoxia (N_2_). The boxplot shows the median, and upper and lower quartiles. The dots indicate the values obtained for each repetition.

There was a significant interaction between anoxia and irradiation dose effects (χ^2^ = 29.968, df = 1, *p* < 0.001, Table 5). Adult males irradiated at 45 Gy in anoxia were on average 5.7 times more fertile than those irradiated in normoxia (*p* < 0.001, Supplementary Table S3; Figure 6). The highest observed difference in fertility was a 14-fold difference between samples irradiated in anoxia versus normoxia. More variability within samples and between technical repetitions were also observed in the anoxia treated groups as compared to the normoxic groups.

**TABLE 5:** Fixed effect of anoxia on adult dose-response.

	Estimate	Std. Error	*z* value	Pr (>|z|)
(Intercept)	4.3298	0.1959	22.105	<2e-16 ***
Dose45Gy	−5.2631	0.1317	−39.954	<2e-16 ***
Normoxia	−1.0435	0.1425	−7.323	2.43e-13 ***
Dose45Gy:rormoxia	−0.8462	0.1546	−5.474	4.39e-08 ***

Signif. codes: 0 “***” 0.001 “**” 0.01 “*” 0.05 “.” 0.1 “ ” 1.

#### 3.4.2 Effect of Irradiation in Anoxia on Flight Ability

**FIGURE 7:**
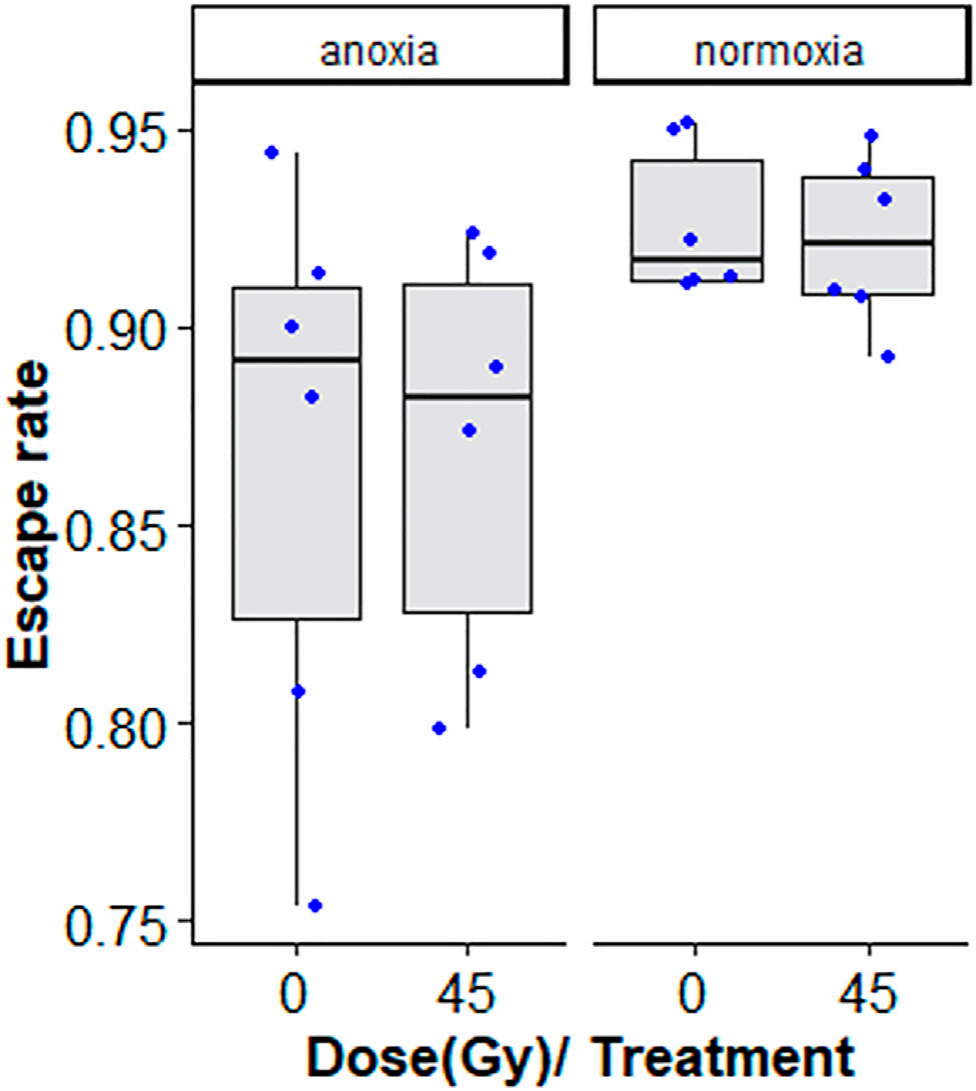
Flight ability of adults treated in nitrogen [non-irradiated controls (N_2_C) and irradiated (N_2i_)] compared to adults in air [non-irradiated controls (O_2_C) and irradiated in air (O_2i_)]. The boxplot shows the median, and upper and lower quartiles. The dots indicate the values obtained for each repetition.

Treatment with nitrogen (anoxia) negatively affected flight ability, regardless of whether irradiated or non-irradiated (GLMM: χ^2^ = 29.642, df = 1, *p* < 0.001, Table 6 and Supplementary Table S4). Irradiation at 45 Gy did not reduce flight ability, neither in the anoxia treatment groups), nor in the normoxic (oxygen) groups (GLMM: χ^2^ = 0.0829, df = 1, *p =* 0.7734, Figure 7; Supplementary Table S4). Again, results were much more variable in groups subjected to anoxia.

**TABLE 6:** Fixed effects of irradiation in anoxia on flight ability.

	Estimate	Std. Error	z value	Pr (>|z|)
(Intercept)	1.922925	0.151896	12.66	<2e-16 ***
Atmnormoxia	0.605234	0.178105	3.398	0.000678 ***
Dose45Gy	−0.00993	0.177462	−0.056	0.955386

Signif. codes: 0 “***” 0.001 “**” 0.01 “*” 0.05 “.” 0.1 “ ” 1.

#### 3.4.3 Longevity Under Mating Stress

**FIGURE 8:**
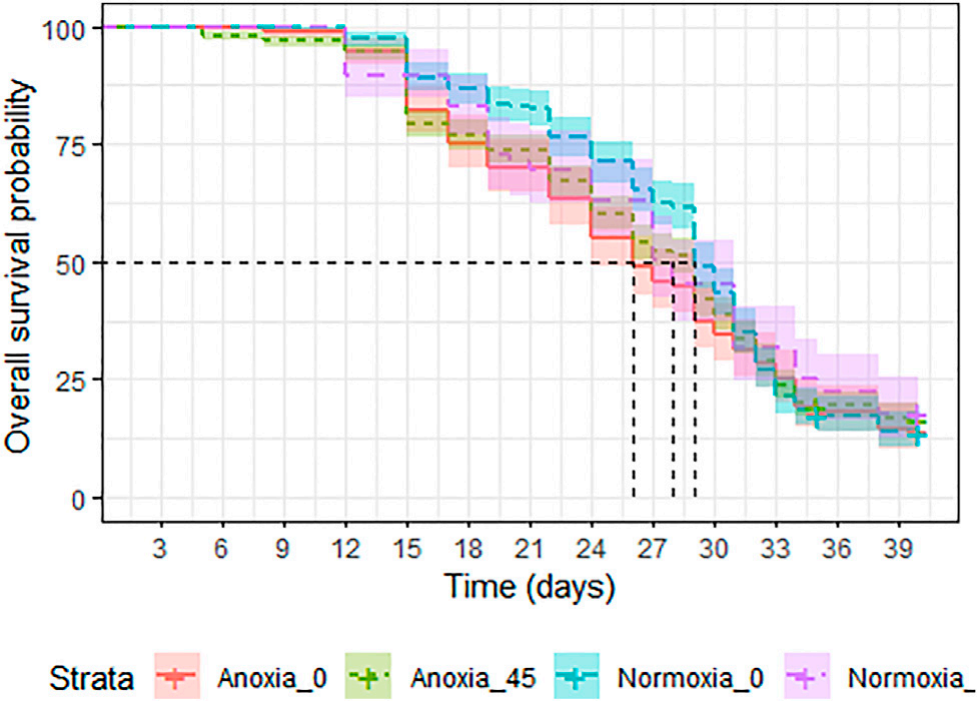
Longevity under mating stress of adult *Aedes albopictus* males irradiated at 45 Gy in either anoxia (N_2_) or normoxia (O_2_) compared to untreated controls.

A significant interaction between dose and atmosphere was observed (LRT: χ^2^ = 19.427, df = 1, *p* < 0.001). However, when comparing each treatment group, anoxia had a negative effect on survival in the non-irradiated groups (Odd ratio = 1.269, z. ratio = 2.652, *p* = 0.04, Supplementary Table S5), but decreased the risk of mortality in the irradiated groups (Odd ratio = 0.631, z. ratio = −3.793, *p* = 0.0009, Supplementary Table S5), when males were caged with females at a 1:1 ratio and were assessed under mating stress (Figure 8). Between the normoxic groups, irradiation with 45 Gy reduced the longevity slightly (Odd ratio = 0.593, z. ratio = −4.146, *p* = 0.0002, Supplementary Table S5). Median survival was 29 (95%CI 29–30) days for untreated controls, and for the goups irradiated in anoxia (95%CI 27–29). Groups irradiated in normoxia and groups treated with only anoxia showed a slightly reduced median survival time of 28 (95%CI 27–31) and 26 (95%CI 27–29) days, respectively (Supplementary Table S6).

#### 3.4.4 Longevity Following High Doses- Males Only

**FIGURE 9:**
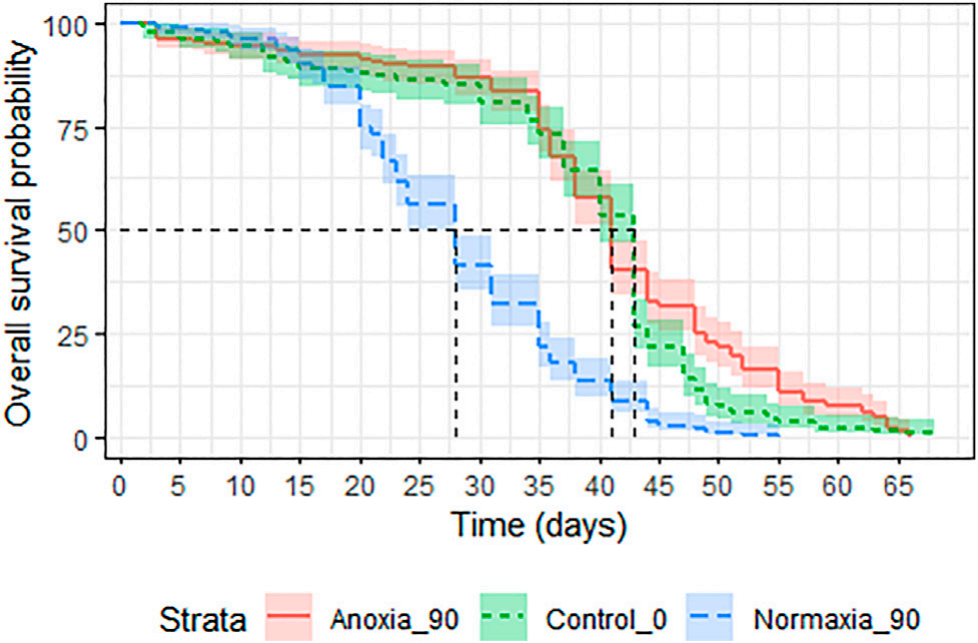
Longevity of adult male *Aedes albopictus* irradiated at high dose (90 Gy) in normoxia (O_2_) or anoxia (N_2_) compared to untreated controls.

Treatment had a significant effect on adult survival (LRT: χ^2^ = 164.9, df = 2, *p* < 0.001). Longevity of males irradiated in anoxia was not affected compared to untreated controls (*p* = 0.054, Table 7; Figure 9) even when the dose was doubled to 90 Gy, wheras males irradiated in normoxia at the same dose were highly negatively impacted (*p* < 0.0001, Table 7; Figure 9). The median survival of the untreated controls and the adults irradiated with 90 Gy in anoxia was 43 (95%CI 40–43) and 41 (95%CI 41-41) days respectively, wheras adults irradiated with 90 Gy in normoxia had a median survival of 28 (95%CI 28-28) days (Supplementary Table S7).

**TABLE 7:** Fixed coefficients of the effects of anoxia with high doses on longevity for males only.

	coef	exp (coef)	se (coef)	z	*p*
Anoxia_90Gy	−0.19949	0.819147	0.103508	−1.93	0.054
Normoxia_90Gy	1.034523	2.813763	0.102299	10.11	0

Signif. codes: 0 “***” 0.001 “**” 0.01 “*” 0.05 “.” 0.1 “ ” 1.

## 4 Discussion

The series of experiments reported here have shown that there are various factors that affect dose response in mosquito adults, which need to be taken into consideration when developing irradiation protocols for adults in the frame of the SIT. These factors not only affect irradiation outcome in terms of sterility levels achieved in the males, but also downstream quality parameters important for male performance once released.

Adult *Ae. aegypti* mosquitoes were slightly more radiosensitive than late-stage pupae. However, one must consider that the younger the pupae, the more sensitive and the more prone to somatic damage (Yamada et al., 2019). In general, *Aedes* pupae, just before emergence seem to be at their most resistant phase to various treatments, such as chilling, desiccation, hypoxic environments and irradiation contrarily to (some) fruit flies, where the pupae are most sensitive on the day before emergence when they are undergoing extensive mitotic divisions for the buildup of the adult organism (Economopoulos, 1977). The dose-response curves for pupae versus adult *Ae. aegypti* corroborate those reported for *An. arabiensis* (Helinski et al., 2006) and *Ae. albopictus* (Du et al., 2019), where adults were slightly more sensitive, but for the most part, the difference was not significant, as was seen in the comparison of late-stage pupae and adult *Ae. albopictus* in the second experiment. The homogeneity of sterility levels within and between adult samples was better than in pupae samples, although pupal age was carefully controlled. Considering that adults do not require higher doses, and the high levels of consistency seen within and between irradiated batches are advantages of irradiating at this stage, in addition to facilitated timing and ease of handling for irradiation exposures.

The next factor that requires scrutiny is the immobilization of adults during bulk irradiation needed for operational programmes. Therefore, assessing the effects of chilling on dose-response in terms of sterility and downstream male performance was essential. Many studies are available in which cold temperatures reduced insect flight ability, or mating competitiveness (Mutika et al., 2002; Shelly et al., 2010; Reynolds and Orchard, 2011; Andress et al., 2014; Diallo et al., 2019), or where cold treatment was used to enhance phytosanitary practices (Gould and Sharp, 1990; von Windeguth and Gould, 1990; Burikam et al., 1992; Follett and Snook, 2013; de Kock and Holz, 2017). However, in these reports, cold treatments were applied separately from the irradiation step, and the direct effect of chilling on dose response was not investigated.

Andress et al., (Andress et al., 2012) found that chilling (3–8°C for 2–6 h) decreased flight ability dramatically in the Mediterraenan fruitfly (*Ceratitis capitata*), whereas Tanahara and Kirihara (Tanahara and Kirihara, 1989), and Reynold and Orchard (Reynolds and Orchard, 2011) found no detrimental effects of chilling on this parameter in the melon fly (*Bactrocera curcurbitae*) and Qeensland fruitfly (*B. tryoni*) respectively. Shelly et al. (Shelly et al., 2010) observed negative effects of chilling on the flight capacity and mating performance of *C. capitata* held at high densities, however, the mating performance was restored after 3 days of recovery. A recovery of host-searching abilities of the parasitoid *Diachasmimorpha longicaudata* following damages from chilling for packaging was also observed after 1–2 days (Cancino et al., 2020). In tsetse flies, Diallo et al. (2019) reported that chilling was one of the main factors negatively affecting the quality of the sterile flies, in terms of emergence rates and flight ability. This corroborated findings of Mutika et al. (2002), who reported a decrease in insemination rates and a dramatic increase in mortality of adult *Glossina pallipides* following low temperature (7 and 4°C)treatment of pupae.

There are numerous other studies that evaluate the effects of temperature on insect quality, however few exist that assessed cold temperature effects during radiation exposures on the irradiation outcome itself. Most studies investigated its direct effects on fertility, or the downstream effects of the combined irradiation and chilling on sterile male quality. For instance, a decrease in survival of adult *G. morsitans* was reported as irradiation dose was increased, and this decline was more pronounced in cohorts irradiated at low temperatures (2°C) (Curtis and Langley, 1972). Langley and Maly (1971) also found chilling to have deleterious effects on adult emergence rates and adult male survival in the fruitfly *C. capitata*, and proposed oxygen-dependent effects of irradiation to be the cause, due to higher oxygen saturation levels at lower temperatures.

Chilling *Ae. albopictus* at 7°C before and during irradiation had no negative impact on longevity when treated as pupae, and only resulted in a marginal reduction in longevity when treated as adults (though not statistically significant). However, the chilling conditions of the present study did slightly reduce the sterility levels achieved as compared to males that did not undergo chilling treatments, but only by a few percent (∼3%). This implies that the cold temperature had some degree of radioprotective effect during the exposure. Here, it was again observed that the variation in sterility levels was higher in and between pupae samples and adult samples were more homogeneous, as was seen with *Ae. aegypti* in the first experiment in this report.

Culbert et al. (2019) studied the effects of chilling in mosquito adults on quality control (QC) parameters, and found that chilling had negative effects on the survival in *Ae. aegypti* and *Ae. albopictus*, where the latter was more sensitive to the cold treatment at all tested temperatures. Contrarily, chilling (at 2,4,6 and 10°C) for up to 8 h had no effect on the survival of *An. arabiensis* for 14 days. Only chilling at 2°C for 24 h resulted in a decrease in longevity in this species. Zhang et al. (2020) found the optimum chilling temperature and duration for *Ae. albopictus* to be 5–10°C for 3 h, resulting in no adverse effects on longevity and mating competitiveness.

Following a similar trend, it was found that chilling did not significantly reduce flight ability, although chilled irradiated groups showed marginally reduced escape rates (though not significant), compared to the unirradiated controls. There was no significant difference between chilled and non-chilled, unirradiated controls, due to a high variation in escape rates in both groups. It is possible that sufficient recovery of chilled adults occurs within the 2 days before the flight test. Significant decrease in flight ability was seen directly after chilling, but near full recovery was observed after 1–2 days in *Ae. aegypti* (Maiga, unpublished results).

The slight reduction in the sterility following irradiation in cold temperatures may be the consequence of the reduced metabolic rates in the mosquitoes, whereas the slight increase somatic damage leading to marginally reduced adult quality in the parameters assessed, and chilling induced damages as reported in other studies (referenced above) may be explained by higher oxygen saturation in the low temperature, leading to an increase in oxygen-dependent effects of irradiation, as proposed by Langely and Maly (Langley and Maly, 1971). In any case, it is known that both radiation damage as well as recovery are temperature-dependent and are both slow in cold temperatures (Sazykina and Kryshev, 2011).

There is a threshold for different insect species at which cold temperatures start to cause negative effects on the organism. For this reason, available studies present either negative or no effects, and seldom positive effects regarding sterile insect quality in the frame of the SIT. The slight radioprotective effects of irradiation in cold temperatures as seen here does not present added value in terms of improving mosquito quality or the SIT, other than its practicality of immobilizing and handling the adult mosquitoes. On the contrary, the degree of chilling and duration is important as it can induce negative effects if not controlled carefully. Therefore, it is worth investigating other methods for immobilization that may improve sterile male quality and irradiation procedures.

Nitrogen can also be used to immobilize mosquitoes for handling and irradiation processes, and its protective effects in insect irradiation have been known since 1947 (Thoday and Read, 1947) and has been widely reported for a variety of insect species.

Hypoxic conditions during insect irradiation have also been shown to often improve insect biological quality, even though higher doses are then needed to reach the desired sterility levels (Economopoulos, 1977; Ohinata et al., 1977; Rananavare et al., 1991). This is because the magnitude of the protective effects seems to be greater for somatic damage than for the induction of sterility (Bakri et al., 2021). For this reason, hypoxic conditions are often used to improve sterile insect quality without reducing their sterility levels (Lance and McInnis, 2005). However, some agents used to create hypoxic environments, such as CO_2_ and N_2_ are reported to have their own negative side effects, some of which disappear again after allowing a period of recovery (Birkenmeyer and Dame, 1970), and some with lasting effects. Other studies on irradiation in anoxia report great improvements on several quality parameters such as longevity, developmental parameters and mating performance (Baldwin and Salthouse, 1959; Baldwin and Chance, 1970; Langley and Maly, 1971; Curtis and Langley, 1972; LaChance and Richard, 1974; Fisher, 1997).

Only few reports exist where irradiation of mosquitoes in nitrogen is described, and effects on quality parameters are assessed. El-Gazzar (1983) exposed *Culex quinquefasciatus* to radiation in a nitrogen atmosphere and showed the reduced effects on sterility induction but found little to no improvement on mating performance. Hallinan and Rai (Hallinan and Rai, 1973) reported that for low doses, nitrogen improved mating competitiveness in *Ae. aegypti*, compared to males irradiated in air, similar to what Terwedow and Asman (Terwedow and Asman, 1977) reported for *Ae. sierrensis*, but none of the publications describe any improvement in other male quality parameters.

Our study also showed that hypoxia protects from O_2_ effects during adult irradiation as was seen in mosquito pupae (Yamada et al., 2019, 2020), but may come with its own negative effects. Anoxia had high radioprotective effects, with up to a 14-fold increase in residual fertility compared to males irradiated with the same dose in normoxia. Anoxia did not have an effect on fertility in unirradiated controls. However, the treatment with N_2_ itself had a negative impact on flight ability. The 2-day recovery time allowed males that were irradiated only, to fully recover flight ability, whereas those treated in N_2_ were unable to recover within this time frame, whether irradiated or not. This implies that the treatment with N_2_ was more important for the reduced flight capacity than the irradiation treatment with 45 Gy. It is possible that a longer recovery time could restore flight ability but storing sterile males for much longer than 2–3 days post irradiation may decrease efficiency in the SIT programmes, where space and extra days of handling are costly.

There was a slight reduction in longevity when adult males were irradiated with 45 Gy in normoxia or anoxia, when they were caged with females at a 1:1 ratio. However, when males were caged alone, and were not subjected to mating stress, those males overdosed with 90 Gy in anoxia survived significantly better than the males irradiated with the same dose in normoxic conditions. We suggest that either 45 Gy is a low enough dose in this species to not see a large effect on longevity [as seen in other reports (Balestrino et al., 2010)], or males irradiated in anoxia are not only more fertile, but also more virile, mating more, and thus slightly reducing longevity in the mixed sex cages, contrary to the results seen at the higher dose, but where females were absent. Males overdosed with 90 Gy in nitrogen lived significantly longer that males irradiated in oxygen and the protective effects of anoxia were clearly observed. To better understand the meaningfulness of longevity studies, it would be important to further examine the effects of the various study designs and variables, such as cage size and adult density, and the inclusion of females (at various ratios) to observe the magnitude of mating induced stress and its effects on male survival. In this study, the males caged alone generally survived more than 2 weeks longer than the males caged with females, suggesting that mating stress has considerable effects on survival and can mask effects of other treatments that are actually the focus of the study.

Although nitrogen had radio protective effects which may preserve fertility and longevity, it seems that treatment with nitrogen in general (with or without the additional radiation exposure) had negative effects on male flight ability, and potentially other parameters which may be the more important factors for mating success in the wild. The full effects of anoxic treatments need to be carefully assessed in field cage mating studies.

There is a need for anesthetics for insect immobilization for facilitating handling. However, many reports have shown that immobilizing agents induce negative side effects (Crystal, 1967; Birkenmeyer and Dame, 1970), the extent of which depends on the sex and age of the insect, as well as the duration and frequency of exposures to the various gases, similar to treatments in cold temperatures. It is therefore necessary to carefully assess these factors and all available options before formulating protocols for mosquito immobilization and handling.

## 5 Conclusion

These experiments gave an initial indication of factors that affect dose-response in mosquito adults, especially in terms of sterility achieved, and downstream effects of chilling and anoxia on selected male quality parameters. Both methods present advantages and disadvantages and affect some quality parameters positively and others negatively. It is important to note that the irradiation dose needs to be adjusted to achieve the desired level of sterility, before considering treatment protocols that could improve sterile male quality for SIT programmes. Additionally, available immobilizing techniques for improved handling need careful evaluation and balance between practicality and potential costs to insect quality to ensure there is a clear benefit before their application in the field.

## Data Availability

The raw data supporting the conclusion of this article will be made available by the authors, without undue reservation.

## Abbreviations

AW-IPM, Area-Wide Integrated Pest Management; DO, dissolved oxygen; FAO, Food and Agriculture Organisation; IAEA, International Atomic Energy Agency; IPCL, Insect Pest Control Laboratory; IS, induced sterility; SIT, sterile insect technique; VAS, ventral air space.
