# Supplementary material for: Effects of Chilling and Anoxia on the Irradiation Dose-Response in Adult Aedes Mosquitoes
Source: Front Bioeng Biotechnol. 2022 May 2;10:856780. doi: 10.3389/fbioe.2022.856780 (PMC9108382; doi:10.3389/fbioe.2022.856780)
Supplement: Supplementary file 1 [file DataSheet1.docx]

**Effects of chilling and anoxia on the irradiation dose-response in adult *Aedes* mosquitoes**

**Yamada H, Maiga H, Kraupa C, Mamai W, Bimbilé Somda NS, Abrahim A, Wallner T, and Bouyer, J**

**Assessing the dose response curve for pupal and adult stages of *Aedes aegypti***

**Supplementary Table 1.** Fixed effects of the dose response curve for pupal and adult stages of *Aedes aegypti*

Fixed effects:

Estimate Std. Error z value Pr(>|z|)

(Intercept) 13.3005 0.7068 18.818 < 2e-16 ***

stagepupae -5.4785 0.8313 -6.591 4.38e-11 ***

log(dose) -4.5646 0.2301 -19.836 < 2e-16 ***

stagepupae:log(dose) 1.7141 0.2684 6.386 1.71e-10 ***

---

Signif. codes: 0 ‘***’ 0.001 ‘**’ 0.01 ‘*’ 0.05 ‘.’ 0.1 ‘ ’ 1

**Effects of chilling on pupae and adult radiosensitivity, and flight ability and longevity in *Aedes albopictus* irradiated as adults**

**Supplementary Table 2*.*** Median survival (±95%Confidence Interval) of chilling and irradiation on adult male *Aedes albopictus*

records n.max n.start events rmean se(rmean) median 0.95LCL 0.95UCL

treatment=chilled_0 36 36 36 36 29.36111 1.5635473 29.5 27 35

treatment=chilled_45 121 121 121 121 28.99174 0.9890802 29.0 28 35

treatment=room_0 40 40 40 40 33.42500 1.7305662 35.5 31 38

treatment=room_45 116 116 116 116 28.81034 1.1020947 29.0 28 35

**Effects of anoxia on adult dose-response*,* flight ability and longevity in *Aedes albopictus***

**Supplementary Table 3:** Odd ratio of the Tukey method for comparing the effect of anoxia on adult *Aedes albopictus* egg hatch

contrast odds.ratio SE df asymp.LCL asymp.UCL null z.ratio p.value

0 anoxia / 45 anoxia 1.93e+02 2.54e+01 Inf 137.6446 2.71e+02 1 39.954 <.0001

0 anoxia / 0 normoxia 2.84e+00 4.05e-01 Inf 1.9688 4.09e+00 1 7.323 <.0001

0 anoxia / 45 normoxia 1.28e+03 1.78e+02 Inf 893.2793 1.83e+03 1 51.342 <.0001

45 anoxia / 0 normoxia 1.47e-02 1.24e-03 Inf 0.0118 1.83e-02 1 -50.148 <.0001

45 anoxia / 45 normoxia 6.62e+00 3.96e-01 Inf 5.6753 7.72e+00 1 31.607 <.0001

0 normoxia / 45 normoxia 4.50e+02 4.31e+01 Inf 351.9718 5.75e+02 1 63.861 <.0001

**Supplementary Table 4.** Odd ratio of the Tukey method for comparing the effect of anoxia on adult *Aedes albopictus* flight ability

contrast odds.ratio SE df asymp.LCL asymp.UCL null z.ratio p.value

**0 anoxia / 45 anoxia 0.967 0.1125 Inf 0.717 1.304 1 -0.288 0.9917**

0 anoxia / 0 normoxia 0.526 0.0630 Inf 0.387 0.716 1 -5.362 <.0001

0 anoxia / 45 normoxia 0.509 0.0785 Inf 0.342 0.757 1 -4.377 0.0001

45 anoxia / 0 normoxia 0.544 0.0972 Inf 0.344 0.861 1 -3.405 0.0037

45 anoxia / 45 normoxia 0.526 0.0630 Inf 0.387 0.716 1 -5.362 <.0001

**0 normoxia / 45 normoxia 0.967 0.1125 Inf 0.717 1.304 1 -0.288 0.9917**

**Supplementary Table 5.** Odd ratio of the Tukey method for comparing the effect of anoxia on adult *Aedes albopictus* survival under mating stress

contrast ratio SE df asymp.LCL asymp.UCL null z.ratio p.value

0 Anoxia / 45 Anoxia 1.193 0.1068 Inf 0.948 1.501 1 1.967 0.2004

0 Anoxia / 0 Normoxia 1.269 0.1142 Inf 1.007 1.600 1 2.652 0.0400

0 Anoxia / 45 Normoxia 0.752 0.0974 Inf 0.540 1.049 1 -2.199 0.1235

45 Anoxia / 0 Normoxia 1.064 0.0815 Inf 0.874 1.296 1 0.816 0.8469

45 Anoxia / 45 Normoxia 0.631 0.0766 Inf 0.462 0.862 1 -3.793 0.0009

0 Normoxia / 45 Normoxia 0.593 0.0748 Inf 0.429 0.820 1 -4.146 0.0002

**Supplementary Table 6*.*** Median survival (±95%Confidence Interval) of anoxia/normoxia and irradiation on male *Aedes albopictus* under mating stress

> summary(fit)$table

records n.max n.start events *rmean *se(rmean) median 0.95LCL 0.95UCL

treatment=Anoxia_0 238 238 238 201 26.37482 0.5803884 26 24 29

treatment=Anoxia_45 739 739 739 602 26.89402 0.3417801 29 27 29

treatment=Normoxia_0 398 398 398 337 28.49420 0.3857225 29 29 30

treatment=Normoxia_45 135 135 135 111 27.64444 0.7738856 28 27 31

**Supplementary Table 7*.*** Median survival (±95%Confidence Interval) of irradiated anoxia/normoxia and non-irradiated male *Aedes albopictus* following high doses- males only

records n.max n.start events *rmean *se(rmean) median 0.95LCL 0.95UCL

treatment=Anoxia_90 217 217 217 217 40.53456 0.9253841 41 41 41

treatment=Control_0 193 193 193 193 38.21244 0.9127159 43 40 43

treatment=Normoxia_90 229 229 229 229 27.93450 0.6535065 28 28 28
